# Differences in intermittent and continuous fecal shedding patterns between natural and experimental *Mycobacterium avium* subspecies *paratuberculosis* infections in cattle

**DOI:** 10.1186/s13567-015-0188-x

**Published:** 2015-06-19

**Authors:** Rebecca M Mitchell, Ynte Schukken, Ad Koets, Maarten Weber, Douwe Bakker, Judy Stabel, Robert H Whitlock, Yoram Louzoun

**Affiliations:** Centers for Disease Control and Prevention, Atlanta, Georgia; Department of Population Medicine and Diagnostic Sciences, Cornell University, Ithaca, NY USA; Department of Bacteriology and TSE, Central Veterinary Institute part of Wageningen UR, Lelystad, The Netherlands; GD Animal Health, Deventer, The Netherlands; Central Institute for Animal Disease Control, Lelystad, The Netherlands; National Animal Diseases Center Ames, 2300 Dayton Avenue, Ames, IA 50010 USA; Department of Clinical Studies, University of Pennsylvania, Philadelphia, PA 19104 USA; Gonda Brain Research Center and Department of Mathematics, Bar-Ilan University, Ramat Gan, Israel

## Abstract

The objective of this paper is to study shedding patterns of cows infected with *Mycobacterium avium subsp. paratuberculosis* (MAP). While multiple single farm studies of MAP dynamics were reported, there is not large scale meta-analysis of both natural and experimental infections. Large difference in shedding patterns between experimentally and naturally infected cows were observed. Experimental infections are thus probably driven by different pathological mechanisms. For further evaluations of shedding patterns only natural infections were used. Within such infections, the transition to high shedding was studied as a proxy to the development of a clinical disease. The majority of studied cows never developed high shedding levels. Those that do, typically never reduced their shedding level to low or no shedding. Cows that eventually became high shedders showed a pattern of continuous shedding. In contrast, cows with an intermittent shedding pattern had a low probability to ever become high shedders. In addition, cows that start shedding at a younger age (less than three years of age) have a lower hazard of becoming high shedders compared to cows starting to shed at an older age. These data suggest the presence of three categories of immune control. Cows that are intermittent shedders have the infection process under control (no progressive infection). Cows that start shedding persistently at a young age partially control the infection, but eventually will be high shedders (slow progressive infection), while cows that start shedding persistently at an older age cannot effectively control the infection and become high shedders rapidly.

## Introduction

Incubation periods and sub-clinical periods of chronic infectious diseases are quite variable in duration, with some individuals becoming ill shortly after infection and some individuals surviving months or years without illness (e.g. Human Immuno-deficiency Virus (HIV) infections [1], Herpes virus infections [2-4], prion induced diseases [5], Mycobacterium bovis [6], Mycobacterium tuberculosis infections [7] and Bovine Leukemia virus (BLV) infections [8]. Time to disease onset is generally assumed to be influenced by a combination of the initial dose received, age at infection, individual immune characteristics and stochastic events influencing progression. However, given poor sensitivity of diagnostic tests for persistent infections, the infected population may represent multiple subgroups, one which controls the infection and is at low risk of progression to disease (e.g. elite controllers in HIV [9]) and a second population that maintains a high probability of infection progression.

An important mycobacterial infection in farm animals is caused by *Mycobacterium avium subspecies paratuberculosis* (MAP). The infection with MAP in cattle often, but not exclusively, occurs in young animals and will lead to long-term persistent infections. In a proportion of MAP infected animals, eventually clinical disease will develop. Clinical signs include chronic diarrhea, weight loss and decrease in milk productivity [10-12]. Eventually, cows would die due to the clinical signs, but usually the owner decides to cull the cows from the herd before the disease progresses to the last stages. Clinical disease due to MAP is called Johne’s disease, after the first veterinarian describing the clinical disease patterns. Although a number of infected animals will show signs of clinical disease, many infected animals do not progress during their lifetime on the farm to a clinical state. These animals may be detected with diagnostic testing during their lifetime, or in post-mortem testing of tissues in the slaughterhouse. Clinical signs are often correlated with high shedding levels [10]. For the sake of consistency, we here use high shedding (above 50 Colony forming units (CFU)/gram feces as a definition of sickness). Note that even if these high shedders do not eventually develop clinical signs, they probably are the main source of infection, and their dynamics are important as such.

The idea of elite controllers, infected individuals with pathogen loads below the detection threshold, have been reported for HIV [13,14] and may be present in the case of MAP. In *Mycobacterium tuberculosis* cases, progression may be triggered in a portion of infected individuals by secondary exposure [15]. Previous work in MAP revealed two initial progression pathways, with or without sufficient early shedding to be detectable by current assays [16].

In this study, we evaluate infection progression in animals followed over the course of their adult lives. ELISA, fecal culture and tissue culture data are available for different subsets of animals in this study. Our objectives include assessing whether all animals are on the same progression pathway, identifying markers for progression, and evaluating whether environment (farm) influenced rate of progression.

Specifically, we plan to address three main issues in the following sections:

A) Can infected cows be divided into two groups: cows that are prone to become high shedders of MAP bacteria and cows unlikely to become high shedders?
B) Given the division into groups; early markers for classification are required. Moreover, in the group with a high shedding probability (high probability of progression to high shedding), is it possible to identify markers for progression. Such Markers can be crucial for management of the infection on dairy farms.
C) Finally, we address the difference in infection progression and shedding patterns in animals after natural versus experimental exposure.

The analysis of MAP within host dynamics relies on different aspects of host immunology and pathogen distribution within hosts. Each of those aspects suffers from different limitations and caveats. While these tests are quite specific, none of the pre-mortem tests are extremely sensitive. Infectious contribution of individual animals can be assessed by fecal culture or PCR. Following death of animals, tissue samples can be used to confirm whether animals are infected with much higher sensitivity than tests available in living animals. However, due to the limited sensitivity of diagnostic tests for MAP infection prior to the emergence of clinical signs, infectious progression patterns are difficult to assess. We here combine a large number of parallel measurements in natural infections to analyze the relationship between shedding patterns and disease progression.

## Materials and methods

### Study populations

Data for this study were gathered from 3 longitudinal field studies, one longitudinal follow up in an experimentally aggregated population and multiple experimental infection trials.

Field study 1 comprised three dairy farms (100, 150, 300 lactating animals per farm T a given time) in the Northeastern US [17]. Animals in study 1 were sampled twice per year by fecal culture and four times per year by ELISA for 7 years after initial farm enrolment. For details of study design, sample collection and preliminary data processing, see previously published work [15,17,18].

Field study 2 followed animals on a single dairy farm with approximately 100 lactating Guernsey breed cattle in Pennsylvania (USA) for a period of 20 years during an intervention program. Details of farm size, MAP prevalence and study design are available in previously published work [19]. Animals in this population were tested semi-annually by fecal culture.

Field study 3 followed animals on 17 Dutch dairy farms (32 to 104 animals per farm with a total of 1072 cows) during a national monitoring program over the course of 3.5 years. Animals were tested by ELISA and fecal culture at 6 month intervals [20].

Naturally exposed animals were aggregated at a research facility for population 4. These animals were either from known infected dams or from known highly infectious environments. Animals were tested by fecal culture at three to six month intervals as well as by immune assays (ELISA) from the time of entrance into the facility. In this population, CFUs were determined on a semi-quantitative scale of +, ++ and +++, representing 1–10, 11–100, and 100+ CFU/sample tube (which is approximately of the order of CFU per gram). This scale was interpreted as 5, 50 and 100+ CFU per animal. For a more detailed description of these cows, see the paper by Karcher et al. [21].

Experimentally infected calves (N = 20) were followed monthly from time of infection to slaughter by fecal culture, ELISA test and either ELISPOT or (other) IFN-g related tests were performed to evaluate infection status. For a detailed description see Langelaar [22].

Throughout this study, only fecal shedding patterns are described. ELISA data were collected but not further analysed in this study. ELISA data are further analysed in a companion paper [15].

### Experimentally infected animals

Shedding patterns from experimentally infected animals were compared to shedding patterns from longitudinally followed naturally infected animals [22]. In most observational studies, the sampling interval was approximately 6 months. Experimentally infected animals had samples collected more frequently than naturally infected animals. To be able to compare these two collection patterns, data from experimentally infected animals were sub-sampled at random. For each 6 month interval, one sample was selected by simple random sampling of all test results within the six month interval.

### Data analysis and statistics

Data from all naturally and experimentally infected animals was aggregated into a single database and analyzed via Matlab v2011a (Mathworks Corporation, Natick, MA, USA). Hazard and survival probabilities were computed using a Cox proportional hazards model, where the events where either first shedding or first high shedding and censoring was based on the absence of future observations (where we assume these cows were removed from the sample).

Survival and hazard curves of high shedding were evaluated by Kaplan-Meier survival analysis [23] with either time in days since birth or time in days since initial shedding as the time scale. To determine whether shedding patterns were dependent on age, animals were categorized by age at initiation of follow-up. Age at first detected high shedding was compared between animals which had a single change in shedding status from non-shedding to shedding (continuous shedders - non-switch) and those that had more than one change in shedding status (intermittent shedders - switch).

Non Parametric methods were used, since the age at first high shedding and the interval between initial and high shedding deviate from a normal distribution. Throughout this paper, shedding of 50+ cfu per gram of faeces is considered high shedding. Low shedders are shedding 1–49 cfu per gram of faeces. When 4 levels of infection were provided (−,+,++ and +++), only +++ was considered high shedding.

Experimental infections are often used to study MAP development and shedding patterns. However, such infections can present substantially different conditions for both environment and infections. These differences can influence the shedding patterns. We compared shedding patterns among experimentally infected and naturally infected animals to evaluate whether experimentally infected animals were representative of natural infections. Shedding patterns were obtained from multiple sources. In order to standardize the different data-sets, we used three shedding levels, as detailed in methods. Using this division, animals were evaluated by the number of changes in shedding state over all available time points. Transitions between non-shedding and shedding states were summed for a total number of “switches” of shedding state. For natural infections in which animals were not followed from birth, animals which were high shedders at their first sampling time point were only included in the analysis if the first sample was taken within their first year in the lactating herd. As all animals would be left-censored at this time point, results should be consistent across study years. Animals which transitioned from non-shedding to high shedding within one sampling interval were assumed to have reached high shedding at the midpoint between the two sampling time points and were considered as continuous individuals. Note that such a scheme can lead to some underestimation of the variance [24].

Survival analysis was performed using the R survival toolbox. The single parameter survival plots was based on the surv and survfit functions implementing a Cox model. The analysis of the farm effect was based on a mixed effect cox model as implemented by coxme [25,26], and the comparison between curves was performed using the survdiff implementing the he G-rho family of Harrington and Fleming [27].

## Results

### Experimental and natural infections have different shedding patterns

**Figure 1:**
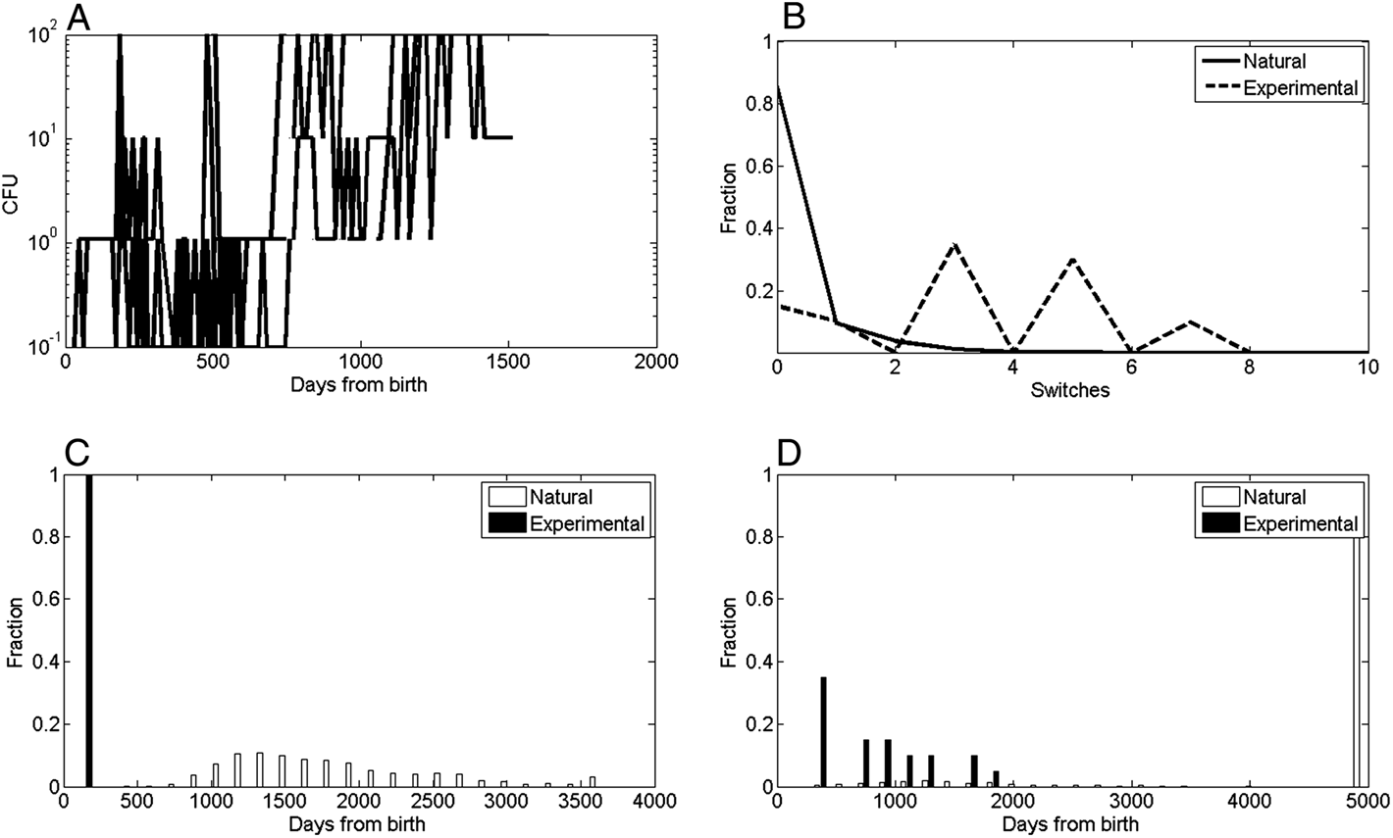
**Comparison of experimental and natural infections. A** - Upper left drawing - typical shedding pattern in experimental infections. Most infected cows switch between low and high shedding, while in naturally infected cows, the shedding level rapidly rise from a non-infected state to high shedding. Each color represents an individual cow. **B** - Upper right drawing. Distribution of switch numbers in naturally infected and experimentally infected cows. The fraction of naturally infected cows with more than 1 switch is less than 5%, while most experimental infections have more than one switch. A switch is defined as an increase followed by a decrease in the shedding level. **C,D** - Lower left and right panels - distribution of times to first shedding (left) and to high shedding (right) from the birth of the cow. Experimentally infected cows shed earlier and reach high shedding earlier than naturally infected cows. The values at 5000 days represent the cows that never reach high shedding.

The shedding patterns of natural and experimentally induced infections differ in both the fraction of cows becoming high shedders and by the dynamics of each cow. In natural infections, becoming a high shedder is a rare and terminal event. Only 7% of the cows ever become high shedders, but once they become high shedders, over 95% of them are culled or die within a year (see Figure 1D for fraction of cows becoming high shedders by age). Note that the expected hazard is much higher than that, since many cows are removed during the study period, or never reached advanced enough age. This is in stark contrast with experimental infections (Gray bars in Figure 1D), where almost all animals eventually become high shedders (85%) (Figure 1D). However, in experimental infections, high shedders can become low shedders again in 95% of the high shedding cows, and then further switch back and forth between low and high shedding (Figures 1A and B).

A similar behaviour is observed for low shedding levels (Figure 1C). Some naturally infected cows do switch between no shedding and low shedding, defined as intermittent shedders. However, naturally infected cows typically switch once (or never), while the experimentally infected cows can switch up to 10 times in the observed sampling period (Figure 1B).

The high number of shedding switches may be associated with the age at which first shedding and first high shedding occur (Figures 1C and 1D). While naturally infected cows typically become shedders after 3 years of life and high shedders after 5 years of life, experimentally infected cows may become shedders within a year and high shedders within another year (Figure 1C).

To summarize, natural infections have a quite simplistic and straight forward shedding pattern composed of a long period of no shedding, followed by a short period of high shedding and death in a small subset. Most naturally infected animals show a pattern of low and intermittent shedding and never progress to high shedding. Experimental infections on the other hand have much more complex shedding patterns with rapid progression and significant fluctuations in shedding levels. These large differences suggest that natural and induced infection represent two different paths to disease. Natural infections represent a population which basically contains the disease, but either external or internal events or characteristics lead to a rapid transition to high shedding in a small proportion of the infected population. Induced infections represent a complex multi-stage progression pattern of infection with a high probability of progression to high shedding. The hazard of becoming a high shedder in naturally infected cows is of the order of 0.0005 per cow per day, with the majority of cows being removed from the herd before ever being high shedders. In contrast, this rate of becoming a high shedder is at least 10 times higher for experimental infections, with over 50% of cows becoming high shedders by the age of three and almost all cows becoming high shedders at some stage of their live.

In the following sections, we focus on natural infection and show that naturally infected cows can be divided into groups with different infection progression patterns. A more thorough discussion of shedding pattern in experimental infection is described in Ganusov and Koets [28].

### Differences in progression among animals which shed consistently and intermittent shedders

We have analysed 3 large field studies and a set of experimental infections. The experimental infections are discussed in detail in the accompanying manuscript [29]. The natural infection were studied in control herds, where cows were only removed when expressing high shedding levels and clinical signs. Detailed statistics of the farms are available in Table 1.

**Table 1:** **Statistics of field studies**

	**Number of cows**	**Number of samples**	**Fraction of positive cows**	**Fraction of high shedders**	**Risk per day**	**Fraction of positive samples**
Study 1	1028	4259	0.586576	0.003891	0.000006	0.012235
Study 2	695	2692	0.726619	0.002878	0.00005	0.027256
Study 3	1072	6112	0.991604	0.182836	0.0002	0.12309

First column is number of different cows, followed by Total number of samples. The following column is the fraction of cows that were ever positive, and the fraction of cows that were ever high shedders. The following columns are the risk per day of becoming high shedder for any cow, and the fraction of all samples that were positive.

**Table 2:** **Frequency of samples with different CFU values in each field study**

	**1**	**2**	**5**	**10**	**22**	**46**	**100**	**215**	**464**	**1000**
Study 1	13	2	2	2	2	1	5	3	0	0
Study 2	31	1	1	1	3	1	1	6	0	0
Study 3	0	0	413	0	0	125	0	0	187	0

Only a small fraction of all naturally infected animals (7%) in our data are ever observed to reach high shedding levels. A larger number of animals have occasional and intermittent low level shedding (15%) Table 2. The observed intermittent shedding pattern can have three possible sources:

- Measurement errors or variation in test sensitivity. CFU tests have a limited sensitivity and cannot detect low levels of bacteria. Many animals studied here had an early period of low shedding levels and are thus probably infected. However, their bacterial load may be below detection. Fluctuations in the test sensitivity or in the conditions of the test may move cows above or below the detection levels. Leading to random observations of positive samples. In these cows, MAP infection is essentially under control of the immune system. These cows have little, if any, clinical signs of the MAP infection.
- In the case that intermittent shedding is real; the periods of shedding may represent random rises in shedding level of a persistently infected cow. Such changes can occur for example through the growth of a large granuloma, eventually resulting in a transition of a large number of bacteria to the gut lumen [29].
- A third possibility is that the short term increase in the shedding level may represent a recent infection that subsequently was cleared or completely controlled by the host. Such true cures of MAP infections have been described in sheep [30] and are suspected to occur in cattle as well. This possibility may represent two distinct phenomena with the same end result: short term shedding due to an infection that was subsequently cleared and/or controlled or short term passive shedding of ingested Map organisms, also denoted a pass-through phenomenon.

**Figure 2:**
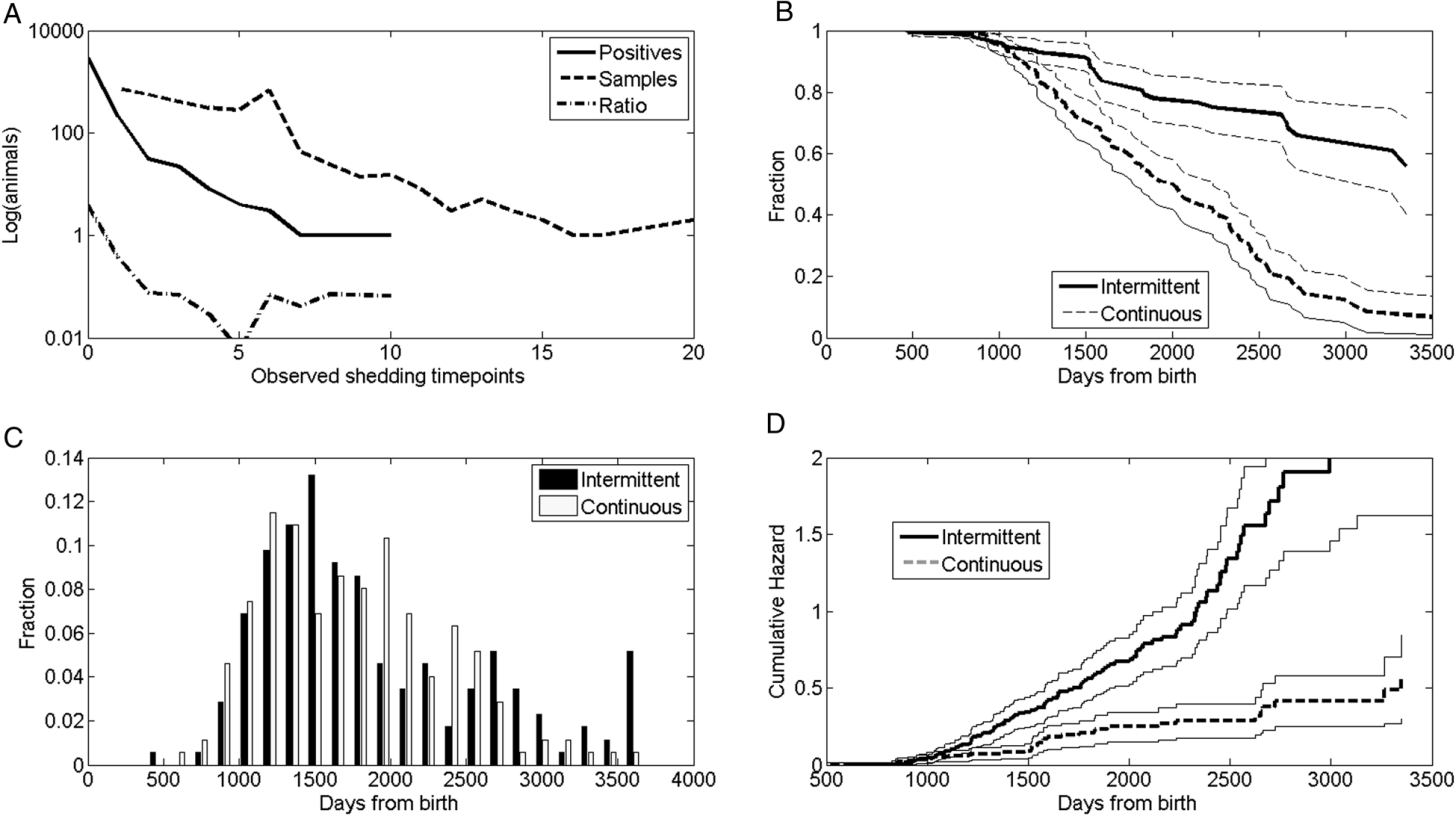
**Comparison of intermittent and continuous shedding. A**. Upper left panel. Histogram of positive sample number (full black line) and total number of samples (grey line). The ratio between the two is not constant showing that positive samples are not a simple artefact of the analysis with a constant probability. **B,D**. Upper right and lower right drawings fraction of cows not reaching high shedding and cumulative hazard of reaching high shedding for intermittent cows and continuous cows. The hazard to become a high shedder is significantly lower in intermittent cows than in continuous cows. **C**. Lower left panel Histogram of time from birth to high shedding for intermittent and continuous shedders. The distribution is highly similar among the two groups.

To disqualify the possibility that intermittent shedders are an artefact of test sensitivity in a population of mostly infected animals, positive tests per individual relative to total tests per individual was plotted for intermittent shedders (Figure 2A). If intermittent shedding was truly an artefact of test characteristics, we would expect the proportion of culture positive samples to remain constant with increasing number of tests per animal. If intermittent represent a population with a distinct shedding pattern or survival characteristics different than non-shedders, the proportion of intermittent would change with number of tests performed. The presented data clearly show that the fraction of positive points is not a constant fraction of samples (*p* < 1.e-10, Chi Square test, 20 degree of freedom).

To test whether intermittent shedding is evidence for a containment of the MAP infection, we separated the populations into cows that have at least one switch (rise to low shedding level, followed by a decrease) and cows that never switched (cows that shed but never went down to no shedding). We denote these populations as intermittent (I) and “continuous shedders” (CS).

We tested the fraction of I and CS cows that never reach high shedding (Figure 2B) and the cumulative hazard of high shedding as a function of time from birth (Figure 2D). The probability of intermittent cows that never reached high shedding (represented as survival in this time-to-event analysis) was significantly higher than the survival of continuous shedders. Conversely, the cumulative hazard of reaching high shedding was significantly lower in cows with an intermittent shedding pattern (less than 5% even after ten years) compared to continuous shedders (*p* < 0.01), suggesting that most of intermittently shedding cows are truly containing the MAP infection and will probably never be high shedding, despite the persistence of MAP infection. When intermittent shedders are compared to continuous shedders, using the G-rho family of Harrington and Fleming, a highly significant difference between the survival of intermittent and continuous shedders is obtained (*p* < 1.e-10). Similarly, when the analysis is repeated to include the farm effect (22 different farms), using a mixed effect cox model (as implemented by the R coxme), a difference between intermittent and continuous shedders is still obtained (*p* < 1.e-10 for the intermittent vs continuous shedding comparison, and *p* < 0.01 for the farm effect). The overall probability per year of becoming high shedder is less 0.0003 + −0.0001 per day (2 standard deviations) per day for intermittent cows, while more than 0.0006+/−0.0001 per day for continuous shedders. Note that the absolute chance per cow is 0.0001 per day, since the majority of cows are neither intermittent nor continuous shedders.

A simple test would be two consecutive samples. Cows shedding once at a low level have a 25% of ever becoming high shedders. Cows shedding twice at low levels have a 48% chance of becoming high shedders, while cows shedding once at a low level and not shedding at the next sample (six month later) have a less than 6% chance of ever becoming high shedders.

### Age effect

**Figure 3:**
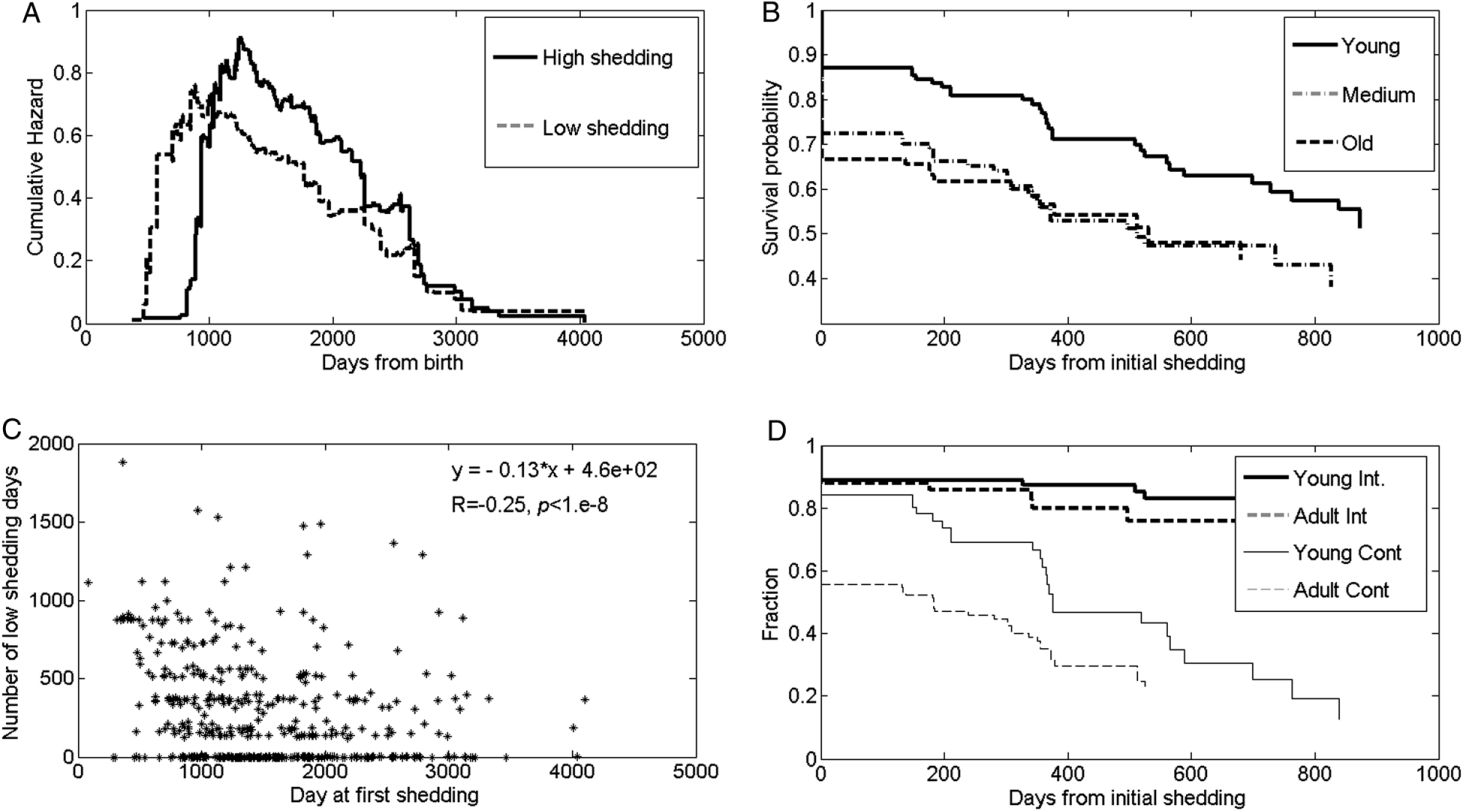
**Effect of age. A**. Upper left panel - hazard of becoming a shedder (gray line) and becoming a high shedder (dark line). The hazard increases rapidly at the age of two, which is first measurement for most cows. The shedding hazard increases significantly before the high shedding hazard. Following the initial rise, the hazard decreases slowly. **B**. Upper right panel. The population was divided into three groups: young cows (less than three years) adult cows (3–5 years), and old cows (above 5 years). Young cows have a much longer time to high shedding than old or adult cows. **C**. Lower left panel - negative correlation between age of onset of shedding and time to high shedding. The line represents a linear regression. The correlation is highly significant (*p* < 1.e-8). **D**. Lower right panel. Fraction not reaching high shedding for young and adult intermittent shedder (thick lines) and for young and adult continuous shedders (thick gray lines). The young continuous shedders survive longer than adult shedders, but less than intermittent shedders.

Beyond, intermittence, age also affects future shedding patterns. The hazard is slowly changing as a function of the cow age (Figure 3A), beyond an initial period, the hazard for shedding increases before the hazard for high shedding. Following an initial period of very low hazard (which could be the result of limited sampling in the first two years of each cow), the hazard peaks to a maximal value, and then slowly decreases. Note that while the initial period of no hazard can be the result of the sampling, the difference between the time to initial high shedding and the time to initial shedding cannot be an artefact of the measurements (Figure 3A).

Following the initiation of shedding, the time it takes to reach high shedding may be affected by the age at first shedding. A clear negative correlation was observed between the age at first shedding and the duration of time until high shedding (Figure 3B) (R = −0.23, *p* < 1.e-8). A difference of 3 years in the initiation of shedding leads to a reduction of 5 months in the time to high shedding.

In order to ensure that this difference is not an artefact of the number of cows in the sample at each age, survival curves and hazard of high shedding were evaluated by Kaplan-Meier survival analysis with time since detection of initial shedding as the unit of time. Animals were categorized by age at initiation of shedding. Three arbitrary age groups were defined (young, medium and old – less than 3 years, 3–5 years, more than 5 years). There was a significant difference between the young infected cows and the other two groups (*p* < 0.05), but no difference between the two older age groups (3–5 and above 5 years) in the time until high shedding (Figure 3C). Younger animals had a significantly lower rate of reaching high shedding compared to medium and old age groups.

Finally, we compared the effect of switching on the time from low shedding to high shedding in young and adult cows. Since there was no difference between medium and old cows, they were combined into a single group. As mentioned above, intermittent cows have a low probability of becoming high-shedders. They thus have a low fraction of high shedders even two years after the first measured positive time point. There is no significant effect of the age of initial shedding on the survival of intermittent animals, suggesting that these animals were able to control the disease. In the continuous shedders, the age of at first shedding has a crucial effect. Cows that start shedding at a young age survive for a much longer time than cows shedding for the first time at and advanced age (Figure 3D) (*p* < 0.05). Note that even at the first shedding point, shedding can be high, thus the fraction of non-high shedders does not start from 1 at time 0.

## Discussion

We have here studied the largest up to now aggregation of shedding histories of MAP infected cows, with over 3500 naturally infected cows and 20 experimental infections. We have compared shedding patterns in natural infections with experimental infections, as well different shedding patterns within natural infections.

Many previous studies were conducted on natural shedding patterns [11,12,15,16,19,31], with the main conclusion being of a short low shedding period before the age of two, followed by a low probability of transition to high shedding. We have here moved beyond the early shedding period and shown that indeed the hazard of becoming a high shedder is of the order of 0.0001 per day per cow, with the majority of cows being removed from the herd before ever being high shedders. In contrast, this rate of becoming a high shedder is at least 10 times higher for experimental infections, with over 50% of cows becoming high shedders by the age of three and almost all cows becoming high shedders at some stage. The difference between experimental and natural infection may originate from higher doses of bacteria in experimental infections or different routes of administration.

Even more important than the lower rate of high shedding, it appeared that naturally infected cows can be divided in two distinct populations. Cows with intermittent shedding, even for one time point, have a significantly reduced hazard of becoming high shedders compared to cows that are continuous shedders. These data strongly suggest that a very large proportion of cows are able to contain the infection and its immune response will result in a persistent infection with little or no pathological consequences. It is expected that these cows will always contain the infection and never move towards high shedding and clinical disease. In a second, much smaller, population of cows the MAP infection cannot be contained and will eventually result in high shedding, clinical disease and eventually culling or death on the farm.

Very few of the intermittently shedding cows do reach high shedding. The few intermittently shedding cows that do reach high shedding do not start with this high shedding at a later age compared to continuous shedding cows (Figure 2C). These non-typical intermittent shedding cows may either be real intermittent, or simply a sampling or diagnostic artefact. In any case, intermittent shedding cows are part of a different population of cows compared to continuous shedding cows. These data would suggest that we have at least two distinct populations of cows, those that contain a MAP infection and those that eventually are not able to contain a MAP infection.

These shedding patterns provide a clear indication for farmers, in case a cow starts shedding. If the cows sheds continuously for some time (even at low levels), it should be considered a high risk animal for the herd. If it stops shedding at some point, then it is highly probable that it will never start shedding again and very likely not progress to high shedding and clinical disease. More precisely, if the animal is faecal culture positive at two subsequent examinations at a 6-month interval, it will become a high shedder with a high probability. If within the same interval, the animal goes back to no shedding; it will have a low probability of becoming a high shedder. This distinction in containing the infection and infection progression is only true for natural infections. Shedding levels in experimental infections fluctuate strongly and these fluctuations are not predictive for future high shedding as almost all experimentally infected cows will become high shedders eventually. Moreover, naturally infected cow that become high shedders, practically never stop being high shedders. Experimental infections are often the opposite with many cows fluctuating from high to low shedding and back.

These considerations strongly suggest that natural and experimental infection follow two different paths to disease. Natural infections are typically dormant until an external or internal event or characteristic drives a change in shedding pattern. When such an event happens, either the immune system controls the infection, as is the case for intermittently shedding cows, or it fails to control it, as is the case for continuous shedders. Experimental infections are characterized by a much more complex pathogenesis with different stages of an active infection and eventual progression to high shedding in virtually all cows.

Some of the intermittent shedders may never be infected, and the observed shedding may be a “pass through phenomenon”. In such a case, we would expect them not to become high shedders. This cannot explain all the intermittent shedding, since intermittent shedders have a higher probability of becoming high shedders than cows never shedding.

The probability to become a shedder or a high shedder is not strongly affected by the age of the cow, beyond an initial period of two years, which may be affected by our sampling method. However, the duration of time a cow will be shedding MAP before it reaches high shedding decreases with the age of the cows at first shedding. When shedding starts later in life, the time until high shedding is significantly reduced. The simplest explanation for this observation would be a weakening of the immune response with age.

These observations taken together shed a clear light on the dynamics of MAP infections. Natural, likely low dose, MAP infections leads to a dormant infection status. The probability to move from this dormant stage to low shedding and eventual high shedding and the duration of the high shedding phase are to be pre-determined by the quality of the immune response. In contrast, high dose experimental challenge infections lead to a rapid transition to an active infectious state, with rapid fluctuations in the amount of free bacteria.

The presented analysis miss the first stage of the infection, since most cows here were not sampled during the first two years of life. It is generally assumed that most naturally infected animals will be infected in their first year of life. Thus, possible correlation between the early shedding properties or properties of the early immune response and the chance to become high shedders were not observed in these data. Another important limitation of the current analysis is the relative low sampling rate (typically every six month) in the natural infections datasets. As a consequence, some rapid fluctuations may in principle be missed. However, if such fluctuations are frequent, we would have observed their signature across the very large number of cows in our study.

Specifically, we have addressed the three main issues outlined in the introduction and:

A) We showed that infected cows can be divided into two groups: cows that are prone to become abundant shedders of MAP bacteria and cows that control the infection.
B) We showed that given MAP infection, the only early marker predicting high shedding that can be detected are the shedding patterns. However, we were not able to find other cow-specific markers for progression to high shedding; suggesting that in the susceptible group transition to high shedding may be a stochastic event [32].
C) Finally, we addressed the difference between experimentally and naturally infected cows. We showed that most naturally infected animals show a pattern of low and intermittent shedding and never progress to high shedding. In small subset natural infections show a long period of no shedding, followed by a short period of high shedding. Experimental infections on the other hand have much more complex shedding patterns with rapid progression and significant fluctuations in shedding levels.

The key finding from this study is the identification of two distinct shedding patterns in naturally infections with MAP in dairy cattle. A very large proportion of MAP infected cows appears to be able to contain the infection and not show any signs of pathology during infection. A much smaller proportion of cows show progression to high shedding and likely clinical disease. Early identification of these animals would be of value in infection control programs.
